# Identification of miRNA from *Porphyra yezoensis* by High-Throughput Sequencing and Bioinformatics Analysis

**DOI:** 10.1371/journal.pone.0010698

**Published:** 2010-05-19

**Authors:** Chengwei Liang, Xiaowen Zhang, Jian Zou, Dong Xu, Feng Su, Naihao Ye

**Affiliations:** 1 Qingdao University of Technology and Science, Qingdao, China; 2 Yellow Sea Fisheries Research Institute (YSFRI), Chinese Academy of Fishery Sciences, Qingdao, China; University of Canterbury, New Zealand

## Abstract

**Background:**

miRNAs are a class of non-coding, small RNAs that are approximately 22 nucleotides long and play important roles in the translational level regulation of gene expression by either directly binding or cleaving target mRNAs. The red alga, *Porphyra yezoensis* is one of the most important marine economic crops worldwide. To date, only a few miRNAs have been identified in green unicellar alga and there is no report about *Porphyra* miRNAs.

**Methodology/Principal Findings:**

To identify miRNAs in *Porphyra yezoensis*, a small RNA library was constructed. Solexa technology was used to perform high throughput sequencing of the library and subsequent bioinformatics analysis to identify novel miRNAs. Specifically, 180,557,942 reads produced 13,324 unique miRNAs representing 224 conserved miRNA families that have been identified in other plants species. In addition, seven novel putative miRNAs were predicted from a limited number of ESTs. The potential targets of these putative miRNAs were also predicted based on sequence homology search.

**Conclusions/Significance:**

This study provides a first large scale cloning and characterization of *Porphyra* miRNAs and their potential targets. These miRNAs belong to 224 conserved miRNA families and 7 miRNAs are novel in *Porphyra*. These miRNAs add to the growing database of new miRNA and lay the foundation for further understanding of miRNA function in the regulation of *Porphyra yezoensis* development.

## Introduction

miRNAs are endogenous non-protein-coding RNAs, approximately 22 nucleotides (nt) in length, that negatively regulate gene expression by complementary binding to the ORF or UTR regions of target messenger RNAs. miRNAs are widespread in animals and plants including unicellular green alga [1], and their expression is highly regulated in a time-dependent and tissue-specific manner across species. Numerous studies have identified miRNAs and their functions in various biological and cellular processes [2], [3], [4]. Generally, their functions have been found to include organ development, cell differentiation and proliferation, cell death and cell apoptosis [3].

To date, a number of miRNAs have been discovered through bioinformatical and/or experimental approaches (cDNA cloning from size-fractionated RNA samples) in various plant species [4], [5], [6], [7], [8], [9], [10]. Most of these miRNAs are conserved across plant families [11]. However, there are species/family specific miRNAs. These specific miRNAs have evolved recently and comprise a smaller fraction of the small RNA library, compared to the conserved miRNAs [12]. Because non-conserved miRNAs frequently accumulate at a lower level than conserved miRNAs, traditional small-scale sequencing often misses the specific-miRNA, the same as most comparative methodologies, particularly when the miRNAs are evolving rapidly [12]. Recently, establishment of high-throughput technologies and deep sequencing analysis has allowed the identification of several miRNAs that are not conserved or are expressed in low levels, such as those found in *Arabidopsis*, rice, poplar, wheat and tomato [13], [14], [15], [16]. However, there is little information available regarding miRNA in algae, except recently published data describing the unicellar green alga, *Chlamydomonas reinhardtii* [1].

The marine red alga, *Porphyra yezoensis* has been proposed as a model marine plant for physiological and genetic studies of seaweed due to its biological and economic importance [17]. To date, no systematic studies of small RNA in red algae have been conducted. The goal of this study was to isolate miRNA from *P. yezoensis*, which will help us to identify the miRNA-based regulatory system of this red alga. High throughput Solexa technology was used to deep sequence the *P. yezoensis* small RNA library and then the sequencing data were analyzed. The miRNAs described here add to the growing database of novel miRNA.

## Results

### Sequence analysis of short RNAs

A cDNA library of short RNAs from *P. yezoensis* was sequenced using the Solexa system. A total of 18,057,942 reads were obtained from the sequencing machine. After removing adaptor/acceptor sequences, filtering out low quality tags and cleaning up the contamination formed by the adaptor-adaptor ligation, 9,738,608 (64.84%) clean reads were obtained, representing 3,425,015 unique sequences. Among the clean reads, 106,085 (1.1%) were found to be similar to known miRNAs. The rest of the sequences were found to be other types of RNA, including non-coding RNA, tRNA, rRNA, snRNA or snoRNA. The numbers and proportions of different categories of small RNAs are shown in Table 1. BLAST searches against *P. yezoensis* EST (above 13,000 ESTs) revealed that 1,089,417 small RNAs (11.19%) matched at least one EST.

**Table 1:** Distribution of small RNAs among different categories in *P. yezoensis*.

Category	Unique RNAs	Percent (%)	Total RNAs	Percent (%)
Total small RNAs	3,425,015	100.00	973,8608	100.00
miRNA	13,324	0.39	106,085	1.09
rRNA	60,472	1.77	639,339	6.56
snRNA	239	0.01	1,803	0.02
snoRNA	246	0.01	610	0.01
tRNA	20,841	0.61	155,483	1.60
Unannotated	3,329,893	97.22	8,835,288	90.72

**Figure 1:**
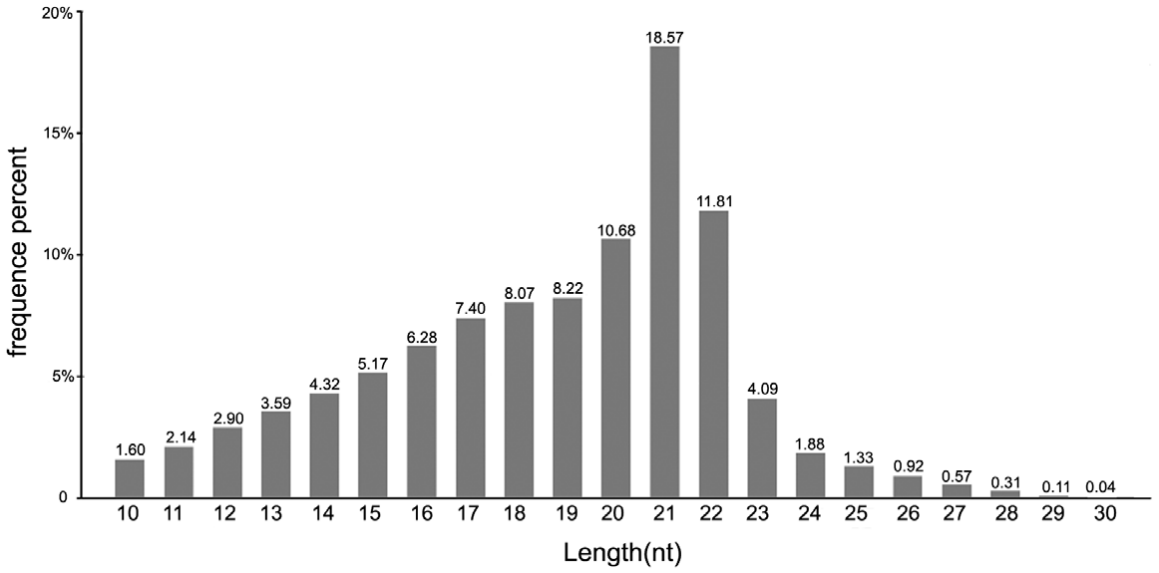
Lengths of unique small RNA sequences in *P. yezoensis*. The occurrences of each unique sequence reads was counted to reflects relative expression level and only small RNA sequences in the range of 10 to 30 nt were considered.

Because small RNAs with known function are commonly 20–24 nt long [16], we analyzed the unique size distribution patterns of small RNAs (Fig. 1). In *Porphyra*, it is observed that the majority of small RNA from *Porphyra* libraries were 22 nt in size (Fig. 1), followed by 22 nt, 19 nt and 20 nt, which is consistent with the typical size of miRNA from Dicer digestion products.

### Known miRNAs and evolutionary conservation

**Figure 2:**
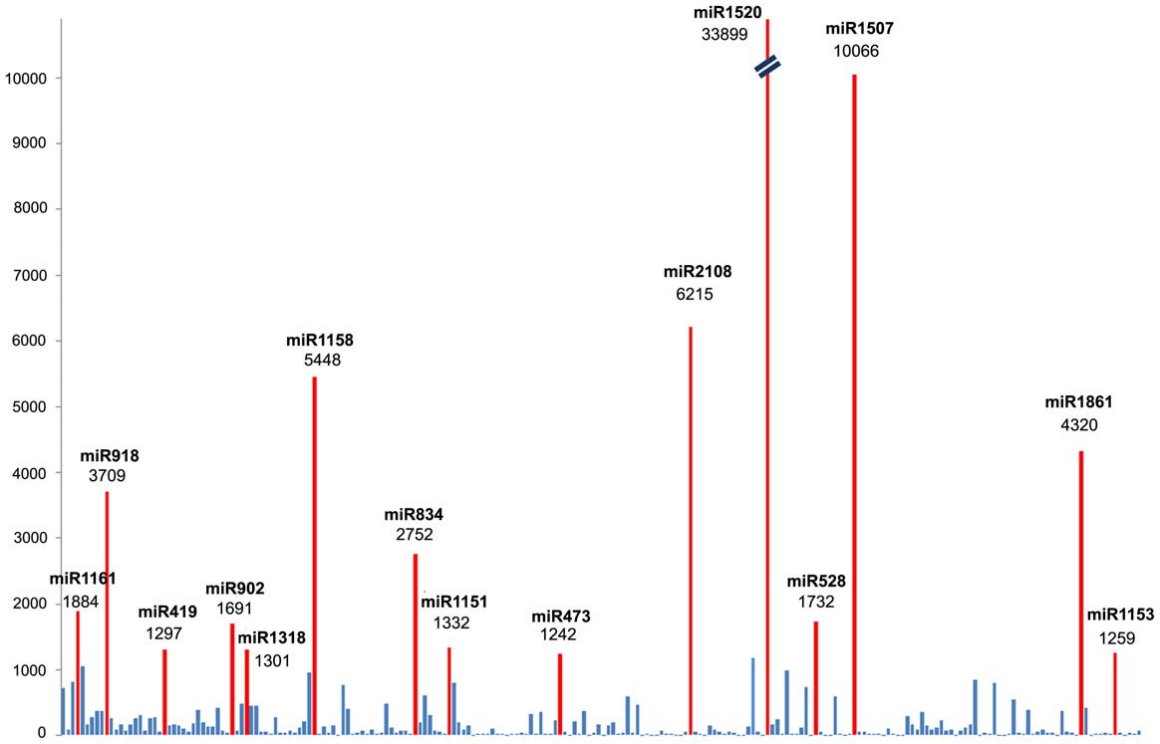
Frequency of known miRNAs in *P. yezoensis*.

To identity the known miRNAs in *P. yezoensis*, we compared our dataset to known miRNAs in miRBase 14.0 (http://www.mirbase.org/). Among the 9,378,608 sequences screened, 13,324 unique sequences were found to be orthologs of known miRNAs from other plant species that had previously been deposited in miRBase (version 14). Allowing one or two mismatches between sequences, these miRNAs represented 224 known miRNA families (Table S1). We analyzed the number of reads for conserved miRNAs and found it has large divergence in the expression frequency among these miRNAs. Among them, 15 miRNAs have relatively more sequence counts, indicating these miRNAs are highly expressed. For example, miR1520 and miR1507 were represented the most frequently in the library with 33,899 and 10,066 copies, respectively (Fig. 2).

To investigate the evolutionary roles of these known miRNA, we performed extensive comparisons against known miRNAs in other plant species, including *Chlamydomonas reinhardtii*, *Physcomitrella patens*, *Selaginella moellendorffii*, *Oryza sativa*, *Arabidopsis thaliana*, *Glycine max*, *Populus trichocarpa*, *Triticum aestivum*, *Brassica napus*, *Medicago truncatula*, *Solanum lycopersicum*, *Brassica oleracea*, *Saccharum officinarum*, *Vitis vinifera*, *Zea mays*, *Sorghum bicolor*, *Gossypium hirsutum*, *Pinus taeda* (Table S2). Among the miRNA sequences obtained from *P. yezoensis*, members in 53 out of 224 families show a lack of conservation of sequence identity when comparing to orthologs in 18 other plant species. Interestingly, *P. yezoensis* and *C. reinhardtii* shared 41 conserved miRNAs (Table S2), and 16 out of 41 miRNAs have no ortholog in other analyzed plants, indicating that these 16 miRNAs were probably involved in regulation of algae-specific processes, for example, adaptation to diverse aquatic environments. In addition, we found 54 miRNAs are conserved in *P. yezoensis* and *Physcomitrella patens*, and 26 out of 54 miRNAs only have orthologs in *P. yezoensis* and *P. patens*. For instance, the target gene of miRNA477 in *P. patens* has several different types of predicted functions (based on protein level sequence homology), including heat-shock protein related, MRP-domain ribosomal protein L29-like and Zinc-finger CCT-domain proteins. miR898 was reported as one of *Physcomitrella*-specific miRNA families which have identified target gene encoding proteins similar to protein kinases [18].

### Novel *P. yezoensis*–specific miRNAs

One of the important features that distinguish miRNA from other small RNAs is the ability of the miRNA flanking sequences to fold-back in a hairpin structure [19]. Because the *Porphyra* genome is unknown, we have to rely on EST sequences to predict the hairpin structure. Our analysis revealed 1,089,417 sequences, each has at least one match in *Porphyra* EST sequences. BLASTN searches against the Rfam database, NCBI GenBank database and miRBase were conducted to remove sequences representing the fragments of non-coding RNAs (rRNA, tRNA small nuclear sequences and small nucleolar RNA), exons, introns and miRNA. The remaining 322,439 sequences containing small RNA sequences that were not associated with any annotation type were mapped to an EST for prediction of novel miRNA candidates.

Our search for new miRNAs revealed that 12 sequences that perfectly matched miRNA ESTs were able to fold into step-loop structures. Of these 12 sequences, seven were considered to be novel miRNA (Table 2). The lengths of these newly identified miRNA sequences were 21 or 22 nt, and they had negative folding free energies ranging from −86.2 to −22.5 kcal mol^−1^ according to Mfold. Additionally, the average free energies of these precursors was found to be about −41.7 kcal mol^−1^, which is much lower than land plant miRNA precursors (−71.0 kcal mol^−1^ in rice, −59.5 kcal mol^−1^ in *Arabidopsis* and −72.4 kcal mol^−1^ in wheat) [20]. To investigate the evolutionary conservation of the putative novel miRNAs, these were searched against the plant genome and ESTs data in KEGG (http://www.genome.jp/kegg/catalog/org_list.html), we found that only one novel miRNA (m0001) has sequence homologous to an unknown gene (CO533805) in *Zea mays*. This observation indicated that putative novel miRNA identified in this study may be species-specific in *P. yezoensis*, i.e. *P. yezoensis*-specific.

**Table 2:** 7 novel miRNA predicted in *P. yezoensis*.

Name	Sequence	Length (nt)	EST no*	Precursor Length(nt)	Engergy kcal mol^−1^
m0001	GACAGCGACGACGACGACGACC	22	AU190053	93	−35.4
m0002	TGGACGCGGCGCTGCGCAAGTC	22	AU192439	251	−86.2
m0003	TACCATGTCGCGGACGGTGTG	21	AU194235	66	−22.5
m0004	TTCGAACCGACCGGGCAATCG	21	AV430788	92	−33.5
m0005	TGTCATGGTCGCTTGGGCACT	21	AV431213	83	−51.2
m0006	TGAGCTGGGAGTTTGGCACCT	21	AV435563	129	−34.1
m0007	TGGCGGCTGGCGTCGTCGAGA	21	AV439175	70	−29.1

*ESTs belonging to same Unigene cluster were not inclued in this table.

### Prediction of miRNA targets in *Porphyra*

**Figure 3:**
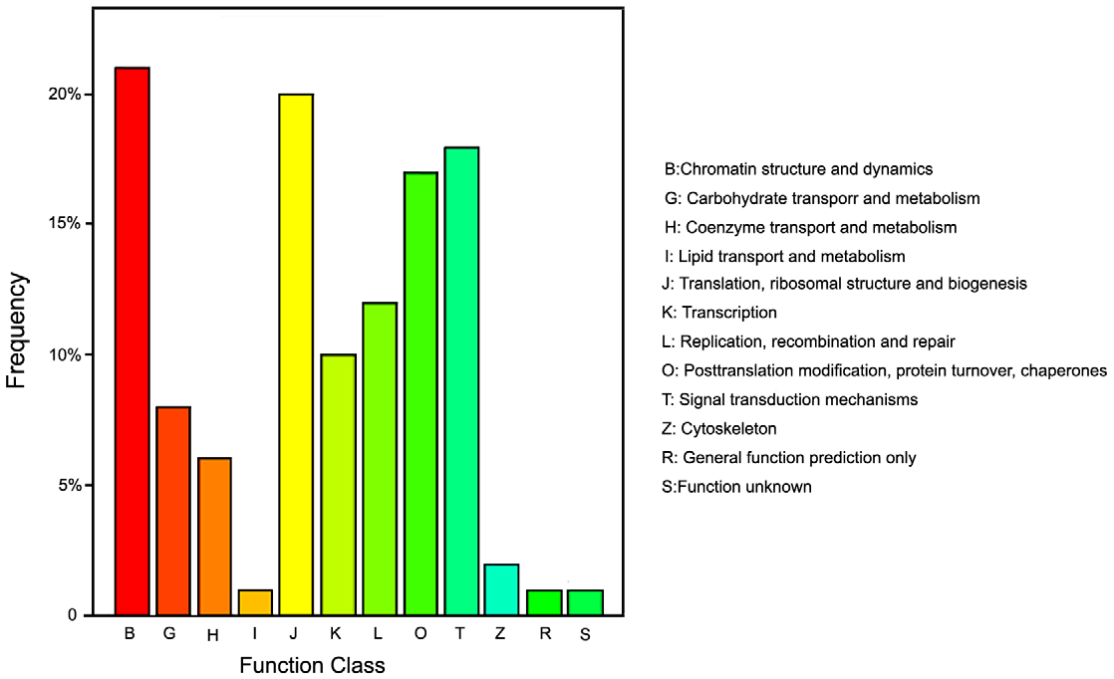
COG function classification of the predicted target genes.

To understand the biological function of miRNAs in *Porphyra*, the putative target sites of the miRNA candidates were identified by aligning miRNA sequences to the ESTs of *Porphyra* while following the rules of target prediction suggested by Allen *et al*. (2005) [21]. The miRNA targets sites of plants have been shown to be primarily located in the coding regions [5], [6]. Consistent with these findings, most of the target sites are located in the coding regions. The putative target genes appear to be involved in a wide variety of biological processes. As shown in Fig. 3, EST sequences corresponding to the putative targets were classified according to the COG database. The predicted target genes included not only transcription factors, but also other genes involved in a broad range of physiological processes. However, we were unable to predict targets for four of the new miRNAs (m001, m002, m003, m005) using the above rules, which may have been due to the limited number of *Porphyra* Est sequences available in the databases. Finally, the predicted targets genes for three of the new miRNAs were novel and had unknown function.

## Discussion

It has been well established that RNAi plays an important role in the silencing of selfish genetic elements and in the formation of heterochromatin in plants, animals and fission yeast [22]. The diversification of small RNA pathways has been demonstrated and the miRNA pathway is known to be a conserved mechanism of gene regulation in eukaryotes [23]. However, this pathway has not been identified in many algae, with the exception of its recent identification in unicellular green algae [1].

The identification of entire sets of miRNAs and their targets will lay the foundation for elucidation of the complex miRNA-mediated regulatory networks that control development and other physiological processes. Most conserved plant miRNAs have been identified by traditional Sanger sequencing. The traditional sequencing method yields longer but less reads. However, the recently developed sequencing technology, Solexa (Illumina), generating shorter reads (up to 35 bp) but yielding 1–3 million reads per sample [24], is very useful to investigate microRNA expression profiles and identify novel miRNAs from new species. Because most miRNAs are only 21–23 nt, we used the Solexa technology to isolate miRNAs from the common seaweed *P. yezoensis*.

The aim of this study was to identify miRNAs present in *P. yezoensis* and miRNAs evolutionary conservation in other plants. When we searched for conserved miRNAs, we found that the *Porphyra* miRNAs have many homologs in higher plants including rice, wheat, *Arabidopsis*, soybean and so on. Also, deep sequencing of the small RNA library allowed identification of the expression levels of each member within a family. Sequence analysis revealed that the relative abundance of certain members within the miRNA families varied greatly, from one to nearly 35,000 copies. Among the conserved miRNAs, miR1520 topped the list of copy numbers with its variant having 33,899 copies but the annotation of its function is still not available. Conversely, miR839, miR1534, miR1445, miR1156 and miR777 appeared to have only one copy (Fig. 2). Our studies also defined these conserved miRNAs shared by other plants, ranging from unicellular algae to higher plants (Table S2), which will provide the opportunity to inspect the evolution of these families during the divergence of plants. 53 miRNA families were *Porphyra*-specific miRNAs. Among them, 17 miRNAs are only conserved in *P. yezoensis* and *C.reinhardtii*. These observations leave open the possibility that these miRNA families could have descended from a common ancestor and diverged or were lost during the diversification of land plants. It is possible that regulatory interactions directed by these algae-specific miRNA are involved in the adaptation to the diverse aquatic environment. Also, as expected based upon previous observations [25], [26], [27], [28], [29], we found some miRNAs conserved among several species. Since these miRNAs had been reported to remain constant during the diversification of land plants [30], we can infer the functions of miRNAs in *P. yezoensis* based on the functions known in land plants. For instance, miR156 directs the cleavage of two SBP box genes which has previously been demonstrated to be a miR156 target [26], [31]. Similarly, miR166 directs the cleavage of the five class III HD-ZIP genes that has been previously been observed to possess miR166 complementary sites [28]. In flowering plants, the miR159/319 family targets subsets of the MYB and TCP transcription factor gene families [32], [33], [34],[35]; miR319 from *P. patens* cleaved a gene with a cyclin domain *in vivo* at the predicted miR319 complementary site [30]. Although the miRNA regulatory pathways are currently unknown, the results of the present study suggest that significant functional differentiation of miRNAs occurs at different developmental stages and some miRNA regulatory mechanisms maybe similar in *Porphyra* and in higher plants.

Despite the lack of genomic sequences, the availability of ESTs helped us to identify seven novel *Porphyra* miRNAs. However, even those miRNAs with miRNA support may not satisfy all of the criteria; hence, some of the novel miRNAs reported here, require further investigation. Most targets of plant miRNAs have a miRNA-complementary site located in their coding regions and occasionally in their 3′ or 5′ UTRs [9], [5], [36], [37]. Consistent with these reports, *Porphyra* miRNAs are predicted to target coding regions. Many of the predicted miRNAs with annotated functions are involved in cellular metabolism and physiological process, whereas transcription factors appear to be under-represented. These findings are consistent with previous studies of *Chlamydomonas*, but contrast many plants in which miRNAs have a remarkable ability to target transcription factor gene families [36]. However, the *Porphyra* genome is not well studied and the EST sequences are limited. As a result, there is no information available regarding the function of a great proportion of protein-coding genes, which makes it difficult to determine if these miRNA targets have functional bias.

The putative novel *Porphyra* miRNAs targeted a number of genes that had no homology to other species' genes, suggesting that these genes may underlie *Porphyra* specific functions. Accordingly, additional studies are necessary to elucidate the role of miRNAs in *P. yezoensis*.

## Materials and Methods

### Strains and culture conditions

Filamentous sporophytes of *P. yezoensis* were grown in seawater medium [38] at 16°C with a 12 h light/12 h dark photoperiod in the laboratory for one week. Then, the materials were collected and cleaned with in sterilized water. After dried with hygroscopic filter paper, the samples were immediately frozen in liquid nitrogen and stored at −80°C before use.

### Small RNA library preparation and sequencing

Total RNA was extracted from the filamentous sporophytes of *P. yezoensis* cultures using Trizol (Invitrogen). The samples were then subjected to 15% denaturing polyacrylamide gel electrophoresis, after which the small RNA fragments of 18–28 nt were isolated from the gel and purified. Next, the small RNA molecules were ligated to a 5′ adaptor and a 3′ adaptor sequentially and then converted to DNA by RT-PCR. Finally, approximately 20 µg products of RT-PCR were sequenced directly using Solexa 1G Genome Analyzer according to the manufacturer's protocols (Beijing Genomics Institute, China) [39]. The sequenced short reads data have been deposited to the Short Read Archive section of GEO at NCBI under accession numbers GSE20534.

### Small RNA analysis

After removing the adaptor/acceptor sequences, filtering the low quality tags and cleaning up the contamination formed by the adaptor-adaptor ligation, the occurrences of each unique sequence reads was counted as sequence tags. Then, there were compared with the sequences of non-coding RNAs (rRNA, tRNA, snRNA, snoRNA) available in Rfam (http://www.sanger.ac.uk/software/Rfam) [40] and the GenBank noncoding RNA database (http://www.ncbi.nlm.nih.gov/) to classify degradation fragments of non-coding RNA. In addition, all sequences were searched for miRNA sequences using miRBase 14.0 [41] to identify the known miRNAs in *P. yezoensis*. Subsequently, we investigated the evolutionary conservation relationship of known miRNAs in *P. yezoensis* and other plants. If a known miRNA in *P. yezoensis* and other plants exhibits homology with less than two mismatches (or >90% homology) reciprocally, it was considered as evolutionary conservation. All small RNA fragments and the identified orthologs of known miRNAs from miRBase were screened from expressed sequence tag (EST) sequences using SOAP 2.0 program [42].

### Prediction of novel miRNA

The prediction of *Porphyra* miRNAs was conducted using criteria that were previously developed for plant miRNA prediction [21]. miRNA precursors have characteristic fold-back structures that can be used to predict novel miRNAs. The prediction was implemented in the Mireap program *developed by the BGI* (*Beijing* Genome Institute). To identify atypical and novel sequences of miRNAs in *P. yezoensis*, we adopted the following strategy. First, candidate miRNA sites were screened out from breakpoints defined by mapping of the small RNAs. Next, a minimal stringent criterion was used to select miRNA candidates, which ensured that the majority of sequences recovered were known miRNAs. Finally, the RNA secondary structure was checked using Mfold [43].

### Prediction of miRNA targets

The putative target sites of miRNA candidates were identified by aligning the miRNA sequences with the ESTs of *Porphyra* using custom Perl script. The rules used for target prediction were based on those suggested by Allen *et al*. (2005) [21] and Schwab *et al*. (2005) [44]. Specifically, the prediction was subject to the following rules: 1) No more than four mismatches between the small RNA and the target (G-U bases count as 0.5 mismatches); 2) No more than two adjacent mismatches in the miRNA: target duplex; 3) No adjacent mismatches in positions 2–12 of the miRNA: target duplex (5′ of miRNA); 4) No mismatches in positions 10–11 of the miRNA: target duplex; 5) No more than 2.5 mismatches in positions 1–12 of the of the miRNA: target duplex (5′ of miRNA); 6) The minimum free energy (MFE) of the miRNA/target duplex should be ≥74% of the MFE of the miRNA bound to its perfect complement. The functional category of obtained EST sequences was annotated against the COG database (http://www.ncbi.nih.gov/COG/) using BLAST program with a cutoff of E value <1e-5.

## Supporting Information

Table S1 The putative miRNA families represented in *P. yezoensis* miRNA pool
(0.05 MB XLS)

Table S2 Evolutionary conservation of known *P. yezoensis* miRNAs in other plants. *P. yezoensis* miRNAs are orthologs of known miRNAs from other plants is indicated with “*”.
(0.05 MB XLS)
